# Enhanced Enzymatic Hydrolysis of Cellulose From Substrate and Indole-3-Acetic Acid Content—During the Fruiting Body Differentiation Stage by Sodium Acetate Addition

**DOI:** 10.3389/ffunb.2021.746313

**Published:** 2021-11-23

**Authors:** Li-juan Hou, Zheng-peng Li, Chang-tian Li, Jin-sheng Lin, Lin Ma, Ning Jiang, Shao-xuan Qu, Hui-ping Li, Yu Li

**Affiliations:** ^1^Engineering Research Center of Edible and Medicinal Fungi, Ministry of Education for Jilin Agricultural University, Jilin, China; ^2^Institute of Vegetable Crop, Jiangsu Academy of Agricultural Sciences, Jiangsu Key Laboratory for Horticultural Crop Genetic Improvement, Nanjing, China; ^3^Key Laboratory of Edible Fungi Resources and Utilization (South), Ministry of Agriculture, Institute of Edible Fungi, Shanghai Academy of Agricultural Sciences, Shanghai, China

**Keywords:** *Volvariella volvacea*, indole-3-acetic acid content, sodium acetate, enzyme activity, mechanism

## Abstract

*Volvariella volvacea*, with high commercial, nutritional and medicinal value, is widely cultivated in tropical and subtropical regions. The effects of supplementation on mushroom yield has been studied. We showed that the optimal application of sodium acetate (NaAc) was spray application of a 0.08% concentration during the substrate mixing stage which could increase yields by up to 89.16% and enhance the enzymatic hydrolysis of cellulose and hemicellulose from the substrate. For most enzymes tested maximum activity occurred during the fruiting body growth and development stage, which led to degradation of the substrate, increasing the available nutrients for mycelial propagation and fruiting body growth and development. Meanwhile, NaAc also significantly increased the indole-3-acetic acid (IAA) content in the early fruiting body development stage of *V. volvacea*, It was observed that IAA promotes not only plant primordium differentiation; but also the primordium differentiation of edible fungi. Furthermore, treatments with three acetate salts had an increase of yield by 30.22% on average. The mechanisms by which NaAc application may improve the yield of *V. volvacea* are discussed.

## Introduction

There are over 200 species of edible and medicinal mushrooms used as functional foods in the worldwide (Kalac, 2013). These mushrooms are rich nutrients, particularly proteins, minerals, and vitamins (Reis et al., 2011; Panjikkaran and Mathew, 2013). *Volvariella volvacea*, belonging to the phylum Basidiomycota (Zheng and Ding, 2013), is one of the most important mushrooms for culinary purposes and is one of five cultivated mushroom species in China (Chang, 1974; Wang et al., 2017). The fruiting body of *V. volvacea* is rich in bioactive metabolites and nutritional qualities that contribute not only to its taste and aroma, but also to its notable anti-coagulant, anti-hypertensive and anti-inflammatory functions (Eguchi et al., 2015).

*V. volvacea* grows fast compared to other mushrooms, with a cultivation cycle of ~14 days (Reyes, 2000). Substrates that are used in cultivating mushrooms have significant effects on their chemical, functional, and organoleptic characteristics (Oyetayo and Ariyo, 2013). Accordingly, nutritional resources, especially carbon (Kitamoto and Tsujiyama, 2015) available to the fungus during development stage are vital to successful fruiting body production (Wang et al., 2012). A balance in the carbon and nitrogen ratio (C/N) of the substrate is important for the total carbon content including recalcitrant cellulose and hemicelluloses (Ryu et al., 2015).

Suitable C/N in substrates regulated by CaCO_3_ could improve *Agaricus bisporus* growth and production (Estrada et al., 2009). In our previous study, applying 0.02% sodium acetate (NaAc) during the water spraying stage improved the yield of *V. volvacea* by 16.25%, increased both carbon and cellulose contents and C/N ratio of the substrate (Hou et al., 2017). Cellulose is the main nutrient responsible for the growth and development of *V. volvacea* (Hou et al., 2017). However, the yield of *V. volvacea* is generally very low and unstable even when supplementation is used compared with the other major cultivated species (Ding et al., 2006). Application of NaAc, simple and low-cost, maximizes the economics for growers of *V. volvacea*. However, little is known for the mechanisms behind yield improvements. Based on our hypothesis that the effects of cellulose content and C/N ratio in the substrate on *V. volvacea* growth are related to lignocellulosic enzymes and hormones, different concentrations of NaAc at different stages was applied for the investigation of the effects on yield and the determination of a standardized application procedure. Lignocellulolytic enzyme activity at different cultivation stages of *V. volvocea*, such as substrate mixing, heavy watering, primordium formation and egg stages was also tested. Meanwhile, the indole-3-acetic acid (IAA) content during the growth and development of fruiting body stage was determined, and the relations of application forms [i.e., anions (Ac^−^) and cations (Na^+^)] with yield, numbers of fruiting bodies, individual mushroom weight and biological efficiency (BE) of *V. volvacea* was explored.

## Materials and Methods

### Strain

*V. volvacea* (strain *V*23), obtained from the China General Microbiological Culture Collection Center (Beijing), (Accession no. CGMCC5.289).

### Substrate Preparation and Inoculation

The experimental method used in this study was similar to that of our previous report (Hou et al., 2017). Specifically, dry cotton waste (95% by weight) purchased locally, was mixed with lime (5% by weight). The mix was pre-moistened with tap water containing 1% lime for 12 h and pH was adjusted to 9.0 before use as substrate (Wu and Li, 2009; Hung et al., 2010). The final moisture in the mix was 65–68%.

The pre-moistened materials were built into piles and left outdoors for 2–3 days covered with white plastic sheets to maintain temperatures for thermophilic decomposers, then turned and mixed when the temperature reached 60°C. The composted substrate was filled onto shelves in a cultivation room with a thickness of ~10–12 cm, covered with white plastic sheeting, and the temperature was maintained at 60–65°C for 5–6 h with heat generated from coke briquettes then slowly dropped to 38°C. *V. volvacea* spawn was cast onto the surface then pressed into the composted and pasteurized substrate at the rate of 300 g/m^2^. Substrate was covered with plastic sheeting after spawning.

### Cultivation, Sodium Acetate Application, and Harvesting

Cultivation of *V. volvacea* was done using methods described previously (Hou et al., 2017), with air temperature set to 30–32°C. Water was sprayed on both test and control substrate 5 days after mycelium colonized surface to induce primordia with a volume of ~1.48 L/m^2^ Concentration of 0.02–0.1% sodium acetate was applied to each test plot as described below. Harvesting techniques were the same as Hou et al. (2017), which started when fruiting bodies reached marketable sizes (egg stage), particularly on Day 10 or 11. First flush normally lasts 3–5 days.

### Experimental Design

Treatments were arranged completely randomly. One flush of mushrooms were harvested whose number was counted and weights were recorded. Yields and biological efficiency (BE) values were calculated after harvesting of the entire first flush. BE was calculated by dividing the fresh weight of mushrooms by the weight of dry substrate used and expressed as a percentage (Royse, 2010). Substrate and fruiting bodies were collected at different growing stages of *V. volvacea* to determine enzyme activity and IAA content respectively. Substrate samples in the mushroom bed using 5-point sampling method, generally take the substrate of the upper layer of about 10 cm, then mix the substrates and liquid nitrogen preservation, transported back to the laboratory and stored at −80, substrate sample were picked randomly at different growing stage (harvesting) for analysis.

### Data Analysis

Data were tested for normality and homogeneity of variance prior to being subjected to statistical analysis. Data on the yield of components (yield (g), number of fruiting bodies, weight per mushroom and BE) were analyzed by one-way ANOVA. All data are expressed as the mean ± SD. All analyses were conducted using the SAS program JMP for practical statistics [SAS Institute (SAS), 2008].

All the chemicals in the experiments were of analytical grade, with the exception of IAA that was standard, which was purchased from Sigma-Aldrich (St. Louis, USA).

### Different Concentrations of NaAc and Yield Components

The concentrations of NaAc as supplement were: 0, 0.02, 0.04, 0.06, 0.08 and 0.1%. At the “spraying water” stage (5 d after inoculation), the spraying volume was set to ~1.48 L/m^2^ and the NaAc solution was applied to each test plot (11.25 kg dry substrate). At the same time, the same volume of tap water was provided to each control group. Each test concentration of NaAc had three replicates in a completely random design as noted above.

### Different NaAc Application Stages and Yield Components

Our previous experiment showed that 0.08% NaAc treatment was the optimum concentration. Therefore, four stages were selected for water spraying. Each treatment had three replicates, and each test plot contained 11.2 kg dry substrate, six treatments in total:

Treatment A: NaAc (1100 ml, 0.08%) at “substrate mixing,” 3 days after natural fermentation of the raw materials, before pasteurization in the growing room;
Treatment B: NaAc (1,100 ml, 0.08%) at “heavy water,” 5 days after inoculation;
Treatment C: NaAc (150 ml, 0.08%) at “primordium,” 7 days after inoculation;
Treatment D: NaAc (150 ml, 0.08%) at “Egg stage,” 9 days after inoculation;
Treatment E: NaAc (0.08%) at stages A–D, each with fixed volume indicated above;
Treatment F: control, tap water only, at stages A–D with the indicated volume for each stage.

### pH Value

The pH values of both treatments and control were measured at the end of the cultivation cycle with pH meter (PHS-3C, Leici, China).

### Cellulolytic Enzyme Activity

During the cultivation process, 2 ± 0.0001 g fresh samples was extracted with 20 ml deionized water in a rotary shaker at 250 rpm for 2 h. The homogenate was centrifuged (12,000 G) at 4°C for 20 min and the supernatant was filtered through filter paper (Whatman No. 1).

Filter paper activity (FP) was assayed according to recommendations by using filter paper as the substrate (Decker et al., 2003). A reaction mixture containing a string of filter paper (Whatman No. 1), 0.8 ml of 50 mM citrate buffer (pH 5.0) and 0.2 ml of appropriately diluted supernatant was incubated at 40°C for 30–120 min. In all assays the release of reducing sugars was measured using the dinitrosalicylic acid reagent method (Miller et al., 1960). One unit of enzyme activity was defined as the amount of enzyme that produced 1 μmol of glucose per minute under the conditions of assay. Endo–β-1,4-glucanase (C_X_) activity comprised 1.5 ml of a 1% carboxymethylcellulose (CMC) solution in 0.05 mol/L citrate buffer (pH 5) and 0.5 ml culture fluid, it was incubated at 50°C for 1 h, it was by the measuring the amount of reducing sugar released from CMC with the Somogyi–Nelson reagents, using glucose as standard. One unit (U) of enzyme activity is defined as the amount of enzyme that produced 1 μmol of glucose per minute under the conditions of assay (Somogyi, 1952). For Exo-β-1,4-glucanase (C_1_) activity comprise 1.5 ml 0.05 mol/L citrate buffer (pH 5), at 45°C for 5 min of preheat, then add 50 mg absorbent cotton (Nanjing Shoude Experimental Equipment Co., Ltd.), it was incubated 45°C for 60 min, absorbance values was determined by the DNS method with glucose as the standard. One unit is defined as the amount of enzyme that produced 1 μmol of glucose per minute (Liu et al., 2011). For β-Glucosidase was determined in 50 mM of sodium acetate buffer (pH 4.8) by measuring nitrophenol released from *p*-nitrophenyl-β-D-glucopyranoside (p-NPG), which is to measure the concentration of p-nitrophenol (p-NP) released from p-NPG catalyzed by the*-*β-Glucosidase after incubation at 50°C for 30 min (Hu et al., 2016). For hemicellulase (HC) activity was determined by measuring the reducing sugars released from xylan as substrates, the reaction mixture for HC activity comprised 0.75 ml of a 0.5% xylan, and 0.25 ml culture fluid, and it was incubated at 50°C for 30 min. U, was defined as the amount of enzyme required to release 1 mg of xylose per 30 min (Shamala and Screekantian, 1986).

### Indole-3-Acetic Acid Content Determination

Freshly-harvested *V. volvacea* mushroom samples were stored in a self-sealing bag placed in liquid nitrogen. 1 to 10 grams of the mushroom sample was weighed and ground into powder with liquid nitrogen. Then, 40 ml of cold methanol (80%) was added to the sample, and the mortar was washed twice with 10 ml of cold methanol (80%), The solution was mixed in a Erlenmeyer flask, sealed with plastic wrap and placed in a refrigerator at 4°C overnight. The solution was shaken ultrasonically for 1 h the next day to fully dissolve the IAA. The extract was suction filtered and evaporated to 2–5 ml under reduced pressure at 40°C. The aqueous solution was transferred to a separatory funnel and then extracted twice with 30 ml of petroleum ether, with the ether phase being discarded. The pH of the aqueous solution was adjusted to pH 8.0 with phosphate buffer (0.2 mol/L) and 3 g of solid PVP (polyvinyl pyrrolidone) was added. The mixture was ultrasonicated again for 1 h and then suction filtered. The pH of the filtrate was adjusted to 3 and extracted with 30 ml of ethyl acetate three times. The ester phases were combined and evaporated at 40°C under reduced pressure until the filtrate was dry. The residue was dissolved in a 10 ml mobile phase solution in a brown volumetric flask and filtered through a 0.45 m microporous membrane filter for analysis (Chen, 2012).

To determine the IAA content, a Nova-Pak column was used (3.9 × 150 mm) with the following parameters: mobile phase V (methanol), 1.0% glacial acetic acid = 30:70; at 0.8 ml/min flow rate, a wavelength of 280 nm, an injection volume of 10 μl and a column temperature of 30°C. An Empower workstation was used to calculate the IAA content (mg/kg) using the following formula: sample peak area × standard concentration × constant volume/(standard peak area × sample weight). To prepare the standard samples, 1.0 ml of acetic acid standard solution (concentration: 98.3 mg/L) was diluted with methanol to a final predetermined concentration (Chen, 2012).

### Effect of Different Anions (Ac^–^) and Cations (Na^+^) on Yield

Three anion salts of acetate and three cation salts of acetate were used as a group to evaluate their effects on yield. Specifically, potassium acetate (KAc), calcium acetate (CaAc), and manganese acetate (MnAc) were used as anion treatment groups. Sodium acetate (NaAc), sodium citrate (NaCi), and sodium borate (NaBo) were used as cation treatment groups. NaAc was used as the control group for both anion and cation treatments. All six treatments were applied at substrate mixing stage, based on the results from previous experiment. In each treatment, 1,100 ml of 0.08% solution was sprayed onto 10.5 kg of dry substrate. Each treatment was conducted in triplicate and arranged in a completely randomized design.

## Results

### Effects of NaAc Concentration on Yield Components of *V. volvacea*

**Figure 1:**
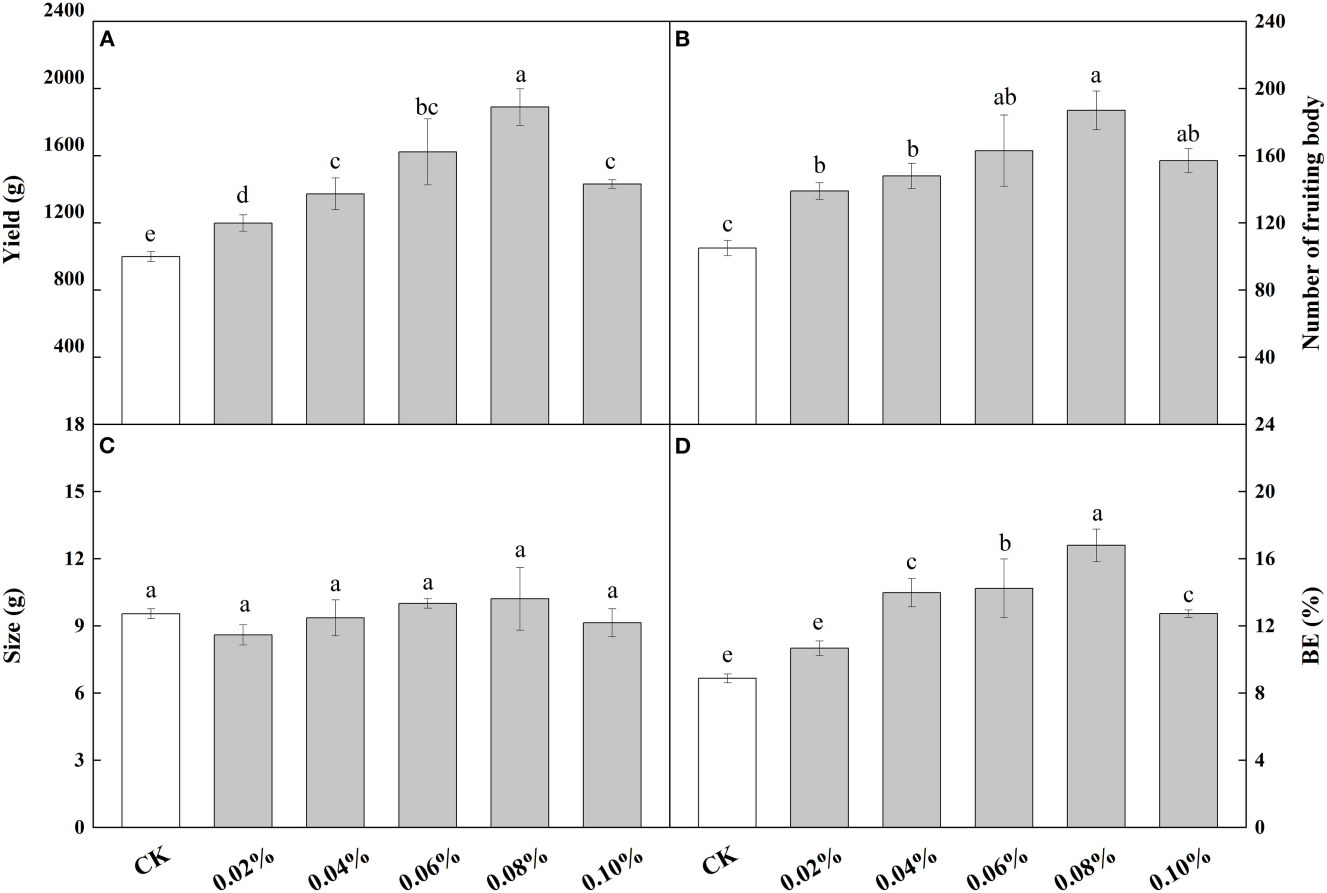
Effects of different concentrations of NaAc on *V. volvacea* yield **(A)**, number of fruit bodies **(B)**, size **(C)** and biological efficiency, or BE **(D)**. Bars indicate the mean ± SD, *n* = 5 at each concentration, while different letters indicate treatments that differed significantly by one-way ANOVA.

When NaAc was sprayed at the heavy water stage, the effects of different concentrations of NaAc on the yield were similar except for treatment E (Figure 1A). In most treatments, the application of NaAc significantly (*p* < 0.05) increased the yield of *V. volvacea* when compared to the control, ranging from 20.05 to 89.16% with the highest yield at 0.08% NaAc. Therefore, NaAc at a concentration of 0.08% was considered the most effective for yield increase.

All concentrations of NaAc treatments produced a larger number of fruiting bodies than that of the control (*p* < 0.05), with increases ranging from 32.38 to 78.09% with the highest fruiting body counts at 0.08% NaAc (Figure 1B).

The size of *V. volvacea* fruiting bodies did not significantly (one—way ANOVA: *p* > 0.05) change with NaAc treatment regardless of the concentration, suggesting that NaAc has little effect on mushroom size (Figure 1C).

Biological efficiency (BE) was similar to yield per plot with a positive linear regression (*p* < 0.05, Figure 1D). The BE increased by 22.22 to 88.89% in the treatments compared to the control. BE was the highest at 0.08% NaAc treatment and then decreased slightly at 0.1%, the highest dose of NaAc applied. These results show that treatments with NaAc can increase BE.

### Effects of Application of 0.08% NaAc at Different Stages on the Yield Components

**Figure 2:**
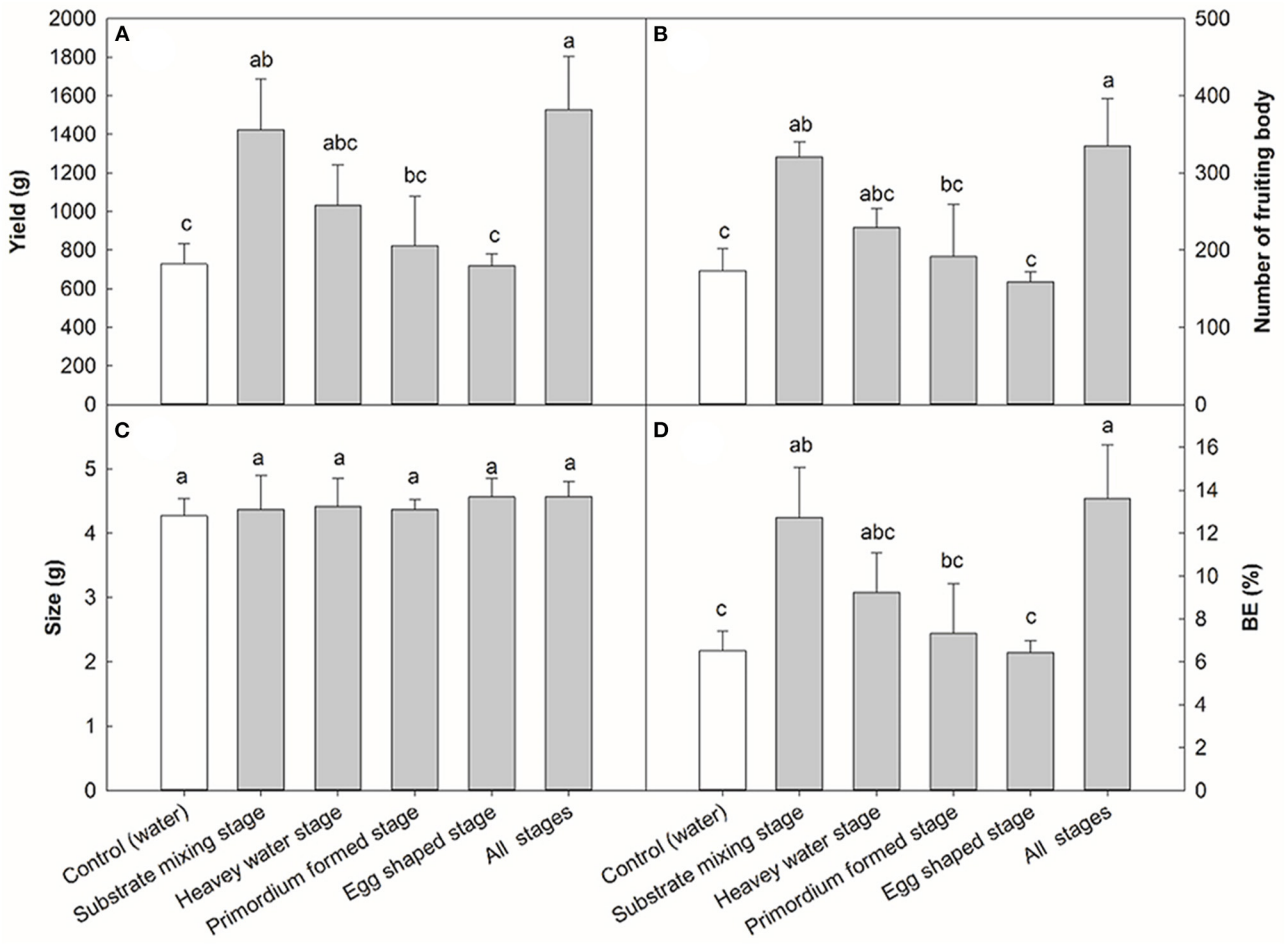
Effects of NaAc sprayed at different stages on *V. volvacea* yield **(A)**, number of fruit bodies **(B)**, size **(C)** and biological efficiency, or BE **(D)**. Bars indicate the mean ± SD, *n* = 5 at each stage, while different letters indicate treatments that differed significantly by one-way ANOVA. Note: heavy stage: 5 days after inoculation the spawn; primordium formation stage: 7 days after inoculation of the spawn; egg stage: 9 days after inoculation of the spawn; all stages: mixed substage stage + heavy stage + primordium formation stage + egg stage.

**Figure 3:**
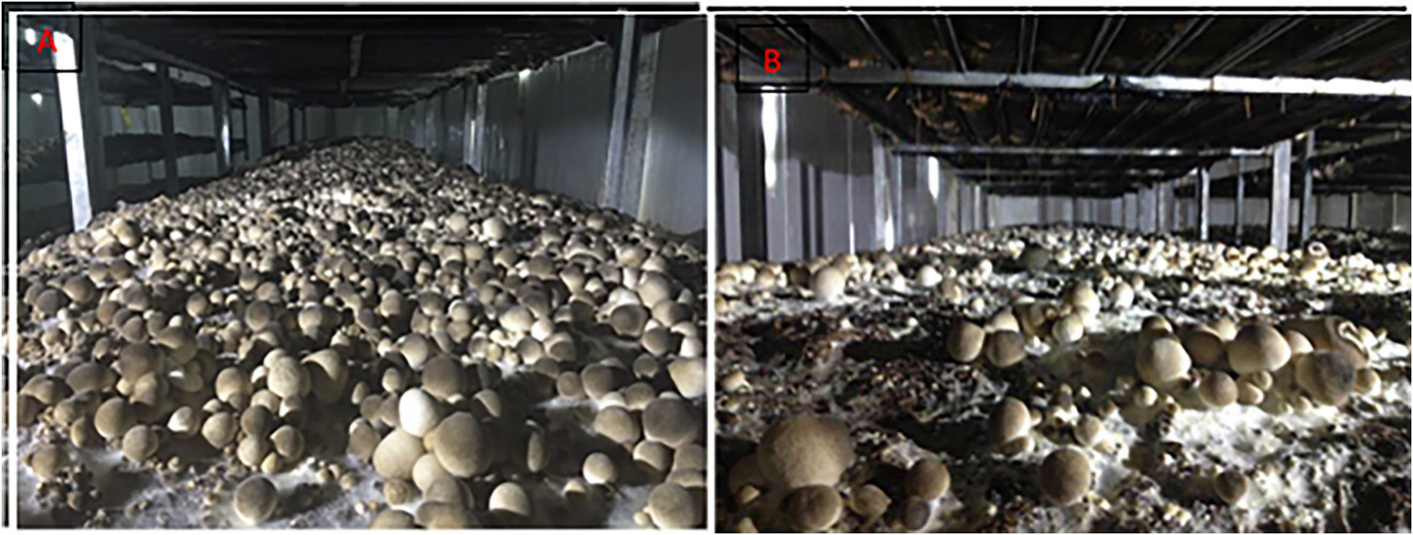
Effects of 0.08% NaAc sprayed at the substrate mixing stage on the fruiting body of *V. volvacea*. **(A)** The fruiting body after application of NaAc. **(B)** The fruiting body of the control.

To determine the optimal stage for NaAc application, the effects of different application stages of 0.08% NaAc was compared, based on the previous experiment shown in Figure 1. The application of 0.08% NaAc at different stages had different impacts on the yield per plot (Figure 2A). When compared with the control (water spraying at all stages), NaAc sprayed at substrate mixing stage increased the yield by 96.74% (*p* < 0.05). The production phenotype of the primordium stage and the egg stage in the farm are shown in Figure 3. Spraying at all stages increased yield by 109.75% (*p* < 0.05) compared to that of the control, but that was not significantly different to the yield when spraying at the substrate mixing stage only (*p* > 0.05). Therefore, spraying of NaAc at substrate mixing stage only is recommended.

The effects of different NaAc spraying stages on the number of fruit bodies displayed a similar trend to that of the yield (Figure 2B). The number of fruiting bodies in the treatment with NaAc at the substrate mixing stage was 321, an increase of 85.55% over the control (173), (*p* < 0.05). When the mushrooms were sprayed with 0.08% NaAc at all four stages, the number of fruiting bodies increased to 335 which was 93.64% higher than that of the control (*p* < 0.05). The application of NaAc at different stages had no significant effect on the mushroom size (g/mushroom) of *V. volvacea* (Figure 2C). Spraying NaAc at the substrate mixing stage increased the BE by 95.69% compared to the control, with an increase of 109.84% (*p* < 0.05) when all stages were sprayed (Figure 2D).

### Effects of the pH Level of 0.08% NaAc at the End of the Cultivation Cycle

At the end of the cultivation cycle, the differences in pH between substrates supplemented with NaAc and that of the control after one flush (Table 1). The pH level of the treatment was 5.93% (0.48) higher than that of the control, indicating that the addition of NaAc increased the pH, although the difference was non-significant. These results are similar to those reported by Schmitz (2002).

**Table 1:** Comparison of the pH values of the treatments and control groups at the end of the cultivation cycle.

**Treatment**	**pH value**	**Mean**	***P* <0.05**
Control	(8.42, 8.47, 8.41, 7.37, 8.54, 7.37)	8.10 ± 023	a
0.08% NaAc	(8.69, 8.63, 8.65, 8.47, 8.53, 8.49)	8.58 ± 0.11	a

*^a^It can be increase the pH value of substrate when spray NaAc, for V. volvacea it like the pH at 8–10 condition*.

### Effects of Application of 0.08% NaAc at Different Stages on Enzyme Activity

**Figure 4:**
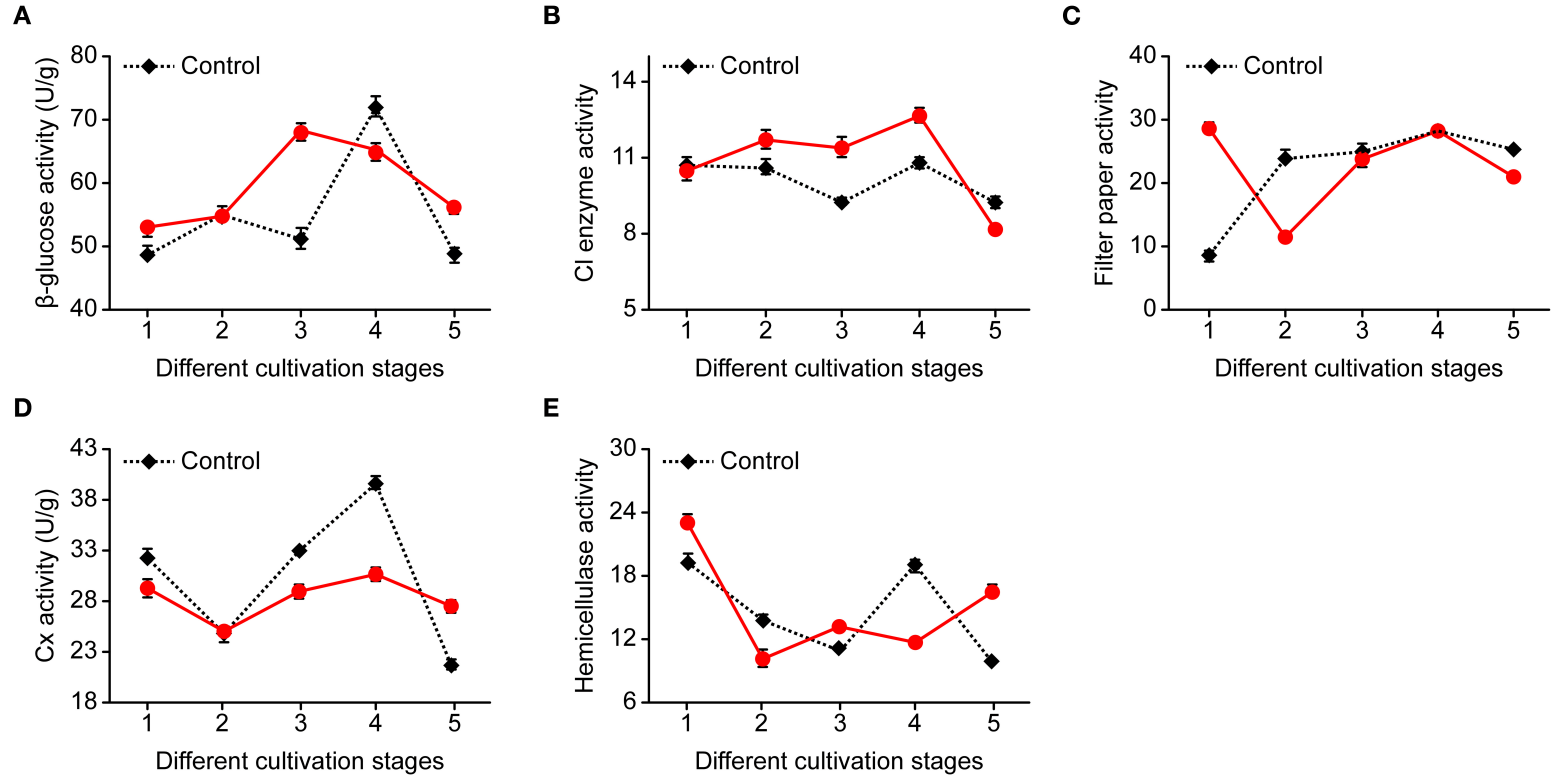
Effects on cellulase activity at different cultivation stages between treatments of 0.08% NaAc and the control. **(A)** β-glucose activity. **(B)** C_l_ activity. **(C)** Filter paper activity. **(D)** Cx activity. **(E)** Hemicellulase activity. 1, heavy water stage; 2, needle stage; 3, primordia formation stage; 4, egg stage; and 5, harvest stage; *n* = 3 at each stage.

The application of NaAc had impacts on cellulase activity. The levels depended on the cultivation stages and enzymes tested (Figure 4). Both the filter paper enzyme and hemicellulase activities were lower at the needle stage when NaAc was applied, compared with the control. FP, Cx and C_1_ had maximum activity during the egg stage in both the NaAc treatment and control groups, while the hemicellulase activity was highest at the heavy water stage in both the treatment and control groups. β-Glucosidase had the highest activity at the primordium stage in the NaAc treatment, whereas the control came at egg stage.

### Effects of Application of 0.08% NaAc at Different Stages on the IAA Content

**Figure 5:**
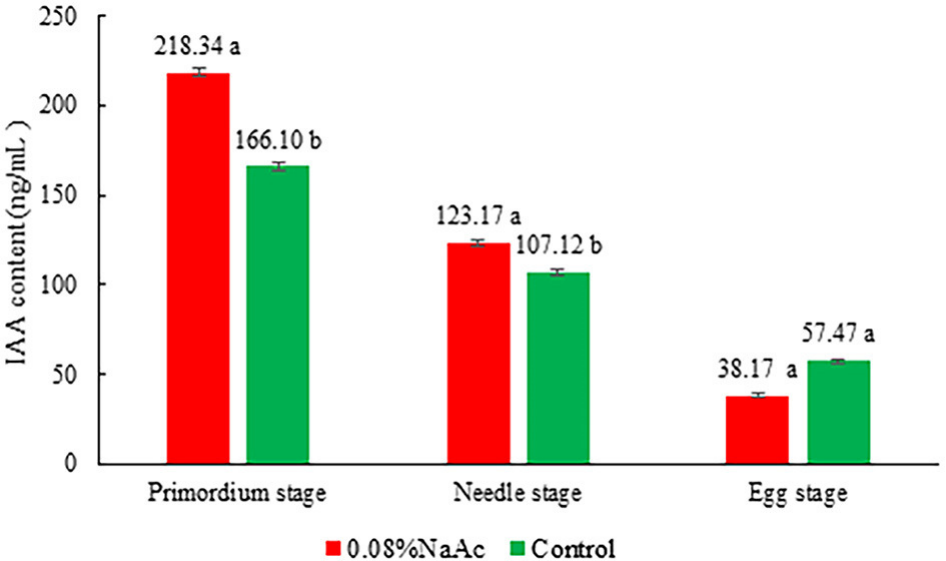
Effects of 0.08% NaAc on the indole-3-acetic acid (IAA) content at different developmental stages. Different letters indicate significant differences by Tukey's *post-hoc* test between the treatment and control groups at each stage, *n* = 3 at each stage.

The application of 0.08% NaAc increased the IAA content by 27.70 and 15.08% (*p* < 0.05) compared with that in the control at primordium stage and button stage, respectively (Figure 5). In contrast, there was no difference in IAA content between the treatment and the control groups at the egg stage (*p* > 0.05).

### Effects of Different Anions (Ac^–^) and Cations (Na^+^) on Yield Components

**Figure 6:**
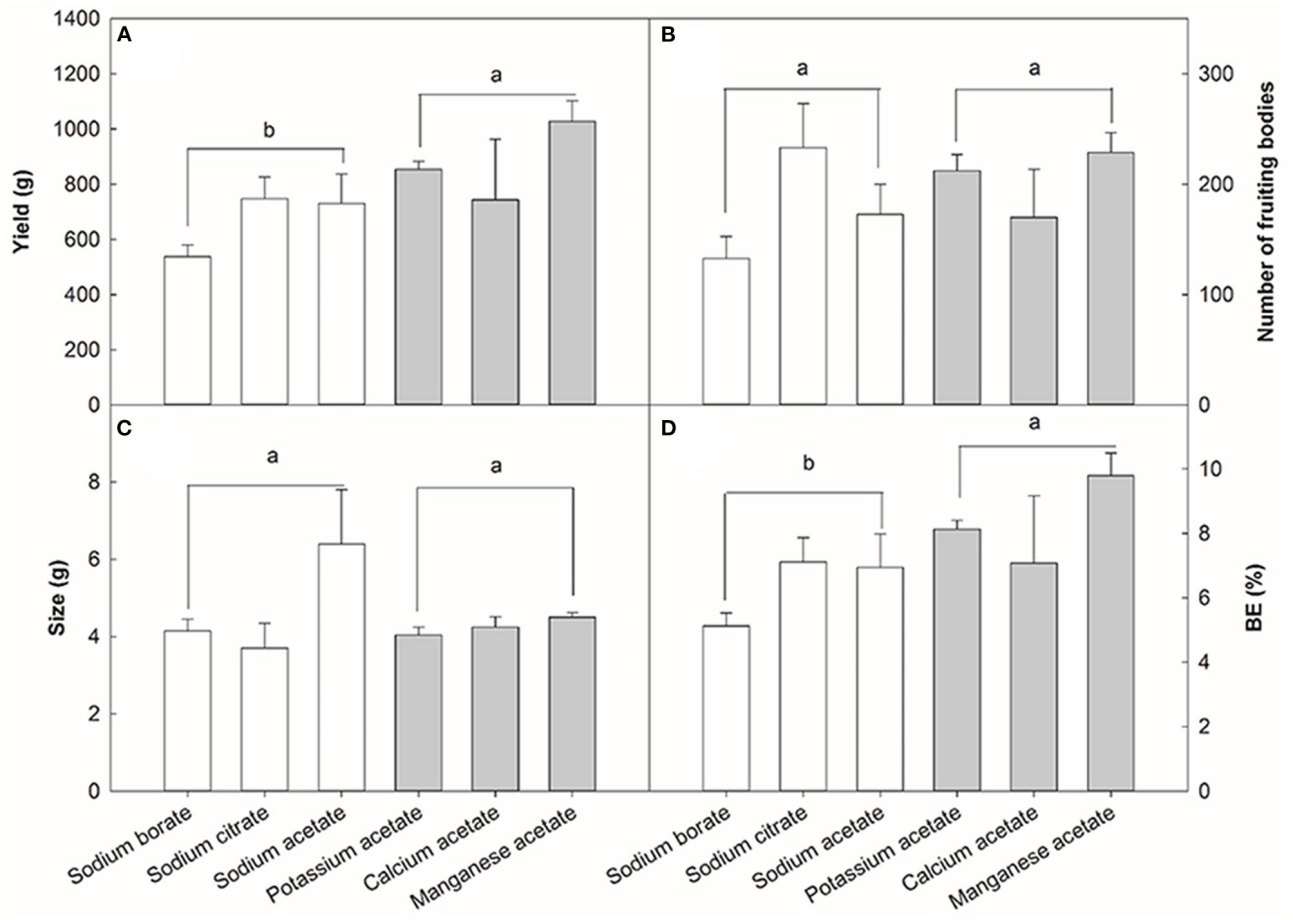
Effect of three anions and cations on the yield **(A)**, number of fruiting body **(B)**, size **(C)**, and BE **(D)** of *V. volvacea*. Sodium acetate, sodium citrate, and sodium borate were used as the cations sources, while potassium acetate, calcium acetate and manganese acetate were used as the anions sources. Sodium acetate was used as an internal control. Bars indicate the mean ± SD, while different letters indicate significant differences by Tukey's *post-hoc* test between anion and cation treatments, *n* = 3 for each treatment.

The previous results showed that the optimal concentration of NaAc was 0.08% and the optimal application stage was the substrate mixing stage. Based on these results, the impact of Na^+^ and Ac^−^ on the yield of *V. volvacea* was investigated. *Three* types of anions and cations were used (Figure 6): KAc, CaAc and MnAc as anions; NaAc, Naci and NaBo as cations. The mean yield of the three anion treatments was 30.30% higher than the mean yield of the three cation treatments (*p* < 0.05, Figure 6A). The treatments with different anions and cations had no significant effects on the number of fruiting bodies (Figure 6B) or mushroom weight (Figure 6C). The BE of different treatments (Figure 6D) had a similar trend to that of the average yield (Figure 6A). The highest BE was achieved in the MnAc treatment group, and the lowest was in the NaBo treatment group. The mean BE of the anion treatments was 30.16% higher than those of the cation treatments (*p* < 0.05, Figure 6D).

## Discussion

To achieve high yields has always been a goal for any grower, especially those of low yields varieties such as *V. volvocea*. Based on our previous work, the application of NaAc can significantly improve the yield of *V. volvacea* significantly (Hou et al., 2017). Three experiments were conducted to explore further results.

According to our results showed that application of 0.08% NaAc during the mixing stage brought a maximized yield of *V. volvacea*, which exhibited the greatest improvement of yield (Figure 3A) compared with that of the control (Figure 3B). The observed improvement in yield as a function on NaAc supplementation may be caused by several factors.

Mushrooms produce a variety of extracellular enzymes that enable them to degrade complex lignocellulosic substrates into soluble substances (Sparling et al., 1982). The growth and fruiting of *V. volvacea* therefore depends on the ability of the fungus to utilize cellulose and hemicellulose from rice straw and other lignocellulosic materials as nutrition. The growth is determined by the mushroom's capacity to synthesize the hydrolytic enzymes necessary to degrade the polysaccharides into low-molecular-weight sugars which can be readily assimilated (Cai et al., 1998). The edible mushroom *V. volcacea* grows naturally on paddy straw which has a relatively low lignin content (Chang, 1974) and does not grow well on substrates with a high lignin content. Different growth and development stages had different cellulase-degrading activity levels. There is a need for enzymatic saccharification of biomass, which is essential to cellulose-degrading enzymes when using lignocellulosic biomass (Donohoe, 2015). Therefore, this study, we focused on cellulase and hemicellulase production.

In our study, the enzymatic activity of HC, C_I_, C_X_ and β-D-glucoside was relatively low during the early mycelial growth stage, and maximum activity occurred during the maturation of the fruiting body. These results were similar to those reported previously in other mushrooms such as *Lentinus edodes* (Gerardo et al., 2016), *Agaricus bisporus* (Wood and Goodenough, 1977) and *Agaricus blazei* (Ni et al., 2001).

Previous studies have reported that the consumption of glycogen storage hyphal cells, trehalose (Hammond et al., 1985) and mycelia proteins (Hammond et al., 1985) are limiting factors for the rapid growth and development of mushroom. Cellulase and hemicellulase had increased activity at the primordium stage after NaAc treatment, which may accelerate degradation of the substrate, meeting the material and energy requirements and increasing the rate of growth and development of fruiting bodies. The results of this study were consistent with this hypothesis.

It was shown that the application of NaAc can increase enzyme activity and a previous study has reported that the application of NaAc can also increase degrade the cellulose and nitrogen content of the substrate (Hou et al., 2017). Therefore, the degradation of nutrients in the substrate is important for *V. vovacea* growth and development.

IAA plays an important role in inducing leaf primordium differentiation, lateral root primordium and flower primordium in many plants (Wu et al., 2015; Lanner, 2017; Mongolli et al., 2017). Chen (2012) previously reported that the highest IAA concentration was in the button stage of *V. volvacea* and that an exogenous IAA solution with a 10^−5^ mol/l concentration could promote the growth of *V. volcace* primordia and increase the yield compared with that of the control.

It was found that the increase in yield was positively correlated with the increase in the number of fruiting bodies and was consistent with the phenomenon of more primordial differentiation on the mushroom bed (Hou et al., 2017). Meanwhile, it was speculated that IAA may promote the primordium differentiation of edible fungi. Application of NaAc also increased the IAA content in the early fruiting body development stage of *V. volvacea*, which indicated that NaAc regulates the IAA biosynthesis pathway. Lv et al. (2018) indicated that auxin might regulate transcriptional levels of the IPT gene to control cytokinin levels and indirectly regulate lateral bud development and growth of *C. appendiculata*. Auxin, as an upstream signal of cytokinin, indirectly regulates plant bud growth and development, and has been obtained in model plants (Müller et al., 2015; Rameau et al., 2015). It is speculated that IAA is related to cell division and primordial differentiation, with increased primordial differentiation leading to increased yield. There is also a direct correlation between greater mushroom production and higher levels of cellulose activity (Arce-Cervantes et al., 2015).

Mushroom survival and multiplication rates depend on several factors that may act individually or have interactive effects (Magan and Aldred, 2008). Chemical composition, water activity, C/N ratio, minerals, surfactant, pH, moisture, nitrogen sources, particle size, inoculum amount, antimicrobial agents and the presence of interactions between microorganisms are chemical, physical and biological factors that are linked to mushroom production (Eira, 2003) and affect mycelia colonization and fruiting potential (Magan and Aldred, 2008). In this study, all cultivation environments and conditions were consistent throughout the entire experiments. When fresh substrate was sprayed with 0.08% NaAc, the pH value was 5.93% (0.48) higher than that of control at the end of the first flush. The addition of NaAc led to an increased pH level during the composting of the substrate. Compared with most commercially cultivated mushrooms such as *Lentinus edodes, Pleurotus eryngii* and *Flammulina velutipes, V. volvacea* thrives in alkaline conditions (Reyes et al., 1998). High pH values are critical for improving the growth, yield and the quality of mushrooms (Khan et al., 1981). When testing the effects of anions (Ac^−^) and cations (Na^+^) on the yield of *V. volvacea*, it was found that those of anions *n* increasing the yield of *V. volvace*a were greater than those of cations. Environmental factors such as water availability, temperature, pH, and their interactions have significant impacts on mycelial colonization and fruiting potential (Magan and Aldred, 2008). Ac^−^ anions are alkaline, maintaining high pH values of the substrate during the fruiting bodies growth and development stage. How the microbial community changes during pasteurization and fermentation on the substrate after NaAc application requires further research. NaAc application might have an effect on microbial fermentation activities during composting which in turn might influence the cellulose content and the C/N ratio in the substrate prior to inoculation.

**Figure 7:**
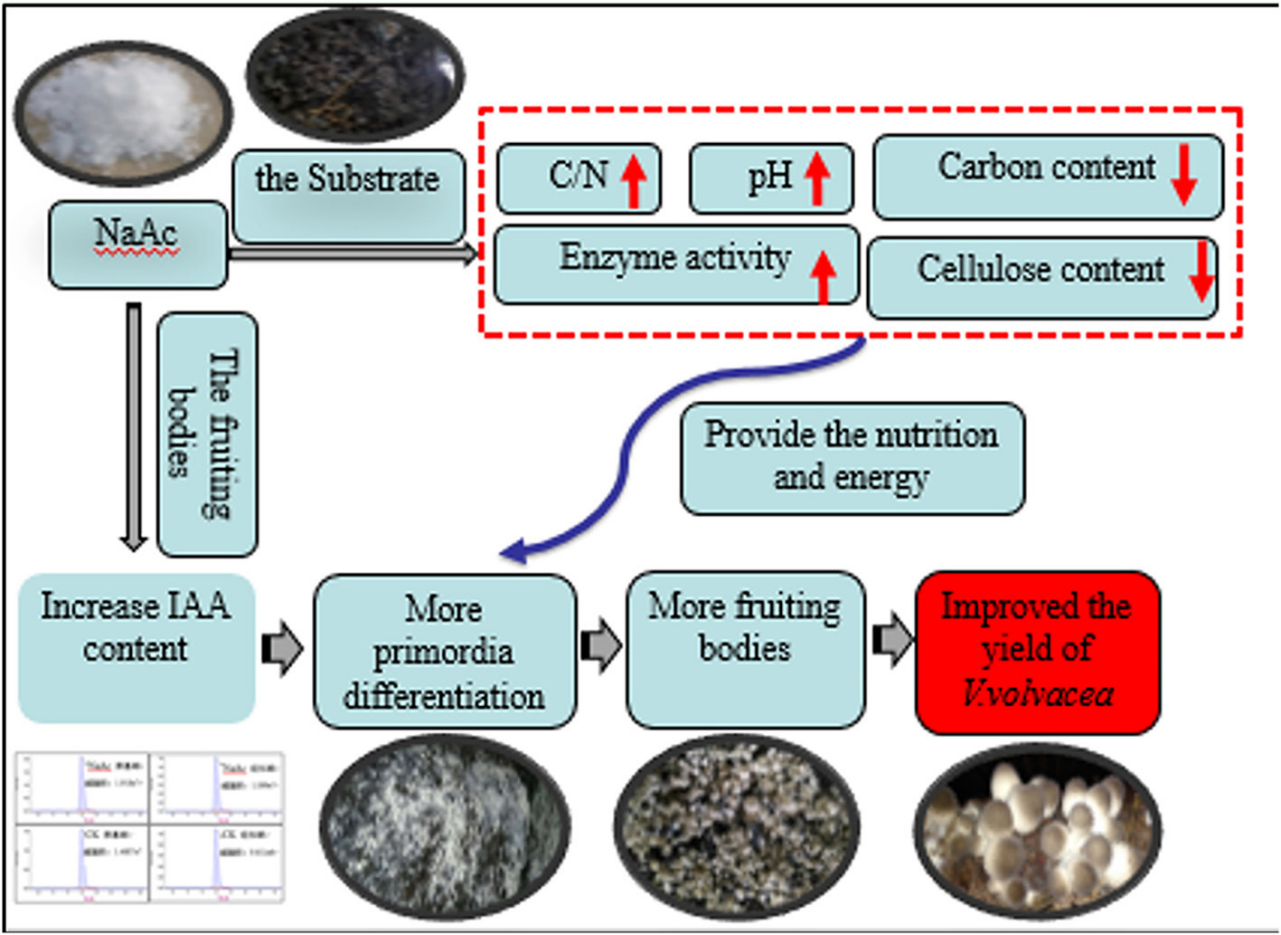
Proposed biochemical mechanisms by which NaAc addition improved the mushroom yield.

This report indicates that increases in total cellulolytic activity and transcription of cellulose genes are related to the rapid expansion of fruit bodies. Thus, by increasing enzymatic activity, the organism can replenish the carbon and energy used in fruiting body development (White ford et al., 2000). Future studies should investigate the related genes and metabolic pathways of fruiting body development, focusing on the effects of auxin on the formation of cytokinins and changes in microbial populations in the substrate at different stages. We propose the biochemical mechanisms by which NaAc addition improved the mushroom yield (Figure 7).

## Conclusions

The addition of NaAc can affect the substrate and fruiting bodies of *V. volvacea* in two main ways. First, increases in the pH, C/N ratio and cellulolytic enzyme activity lead to degradation of the substrate, increasing the nutrition available for mycelia propagation and fruiting body growth and development. Second, the application of NaAc can also increase the IAA content in the early fruiting body development stage of *V. volvacea*. We speculated that increased IAA content causes primordia differentiation, thereby promoting an increase in the number of fruiting bodies.

## Data Availability Statement

The original contributions presented in the study are included in the article/supplementary material, further inquiries can be directed to the corresponding authors.

## Author Contributions

L-jH, C-tL, and YL conceived the study. L-jH, Z-pL, J-sL, and NJ conducted the experiments. LM, S-xQ, and H-pL contributed to sampling and data interpretation. L-jH and YL drafted the manuscript. All authors contributed finalizing to the manuscript.

## Funding

This research was funded by the Applied Basic Research Programs of the Science and Technology Commission Foundation of Jiangsu Province (Grant No. BK20140742), the Open Project Fund of Engineering Research Center of Edible and Medicinal Fungi (SYJ2019001), and the Ministry of Education for Jilin Agricultural University and China, Earmarked Fund for Modern Agro-industry Technology Research System (Grant No. CARS20).

## Conflict of Interest

The authors declare that the research was conducted in the absence of any commercial or financial relationships that could be construed as a potential conflict of interest.

## Publisher's Note

All claims expressed in this article are solely those of the authors and do not necessarily represent those of their affiliated organizations, or those of the publisher, the editors and the reviewers. Any product that may be evaluated in this article, or claim that may be made by its manufacturer, is not guaranteed or endorsed by the publisher.
